# Supplementary material for: Glutamate indicators with increased sensitivity and tailored deactivation rates
Source: Nat Methods. 2025 Dec 23;23(2):417–25. doi: 10.1038/s41592-025-02965-z (PMC12904790; doi:10.1038/s41592-025-02965-z)
Supplement: Supplementary file 1 — Supplementary Figures 1–4 and Tables 1 and 2. [file 41592_2025_2965_MOESM1_ESM.pdf]

nature methods

Supplementary information https://doi.org/10.1038/s41592-025-02965-z

Glutamate indicators with increased sensitivity and tailored deactivation rates

In the format provided by the authors and unedited

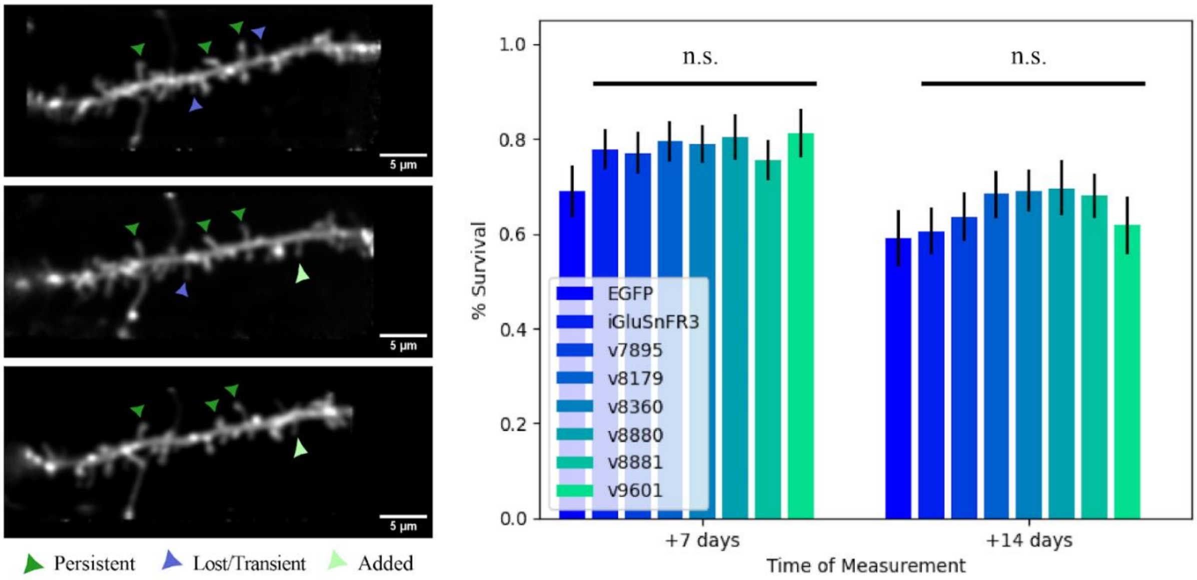

Supplementary Figure 1: Spine turnover of V1 neurons expressing iGluSnFR variants

Left, example images over 3 measurement days at 1-week intervals, for a v8880-expressing neuron, demonstrating persistent, lost, and added spines. Right, Surviving fraction of spines over 3 measurement days at 1-week intervals for the iGluSnFR variants screened *in vivo*, and membrane-tagged EGFP control. Mean +/- Binomial standard error. N= [246 EGFP; 257 iGluSnFR3; 260 v7895; 163 v8179; 221 v8360; 178 v8880; 101 v8881; 142 v9601] spines at initial timepoint. A log-rank test (Kaplan-Meier model) was used to test total survival curves of each variant against EGFP. Chi-squared tests were performed to test the individual measurements taken at +7 days and +14 days against corresponding EGFP measurements. All p-values >0.05.

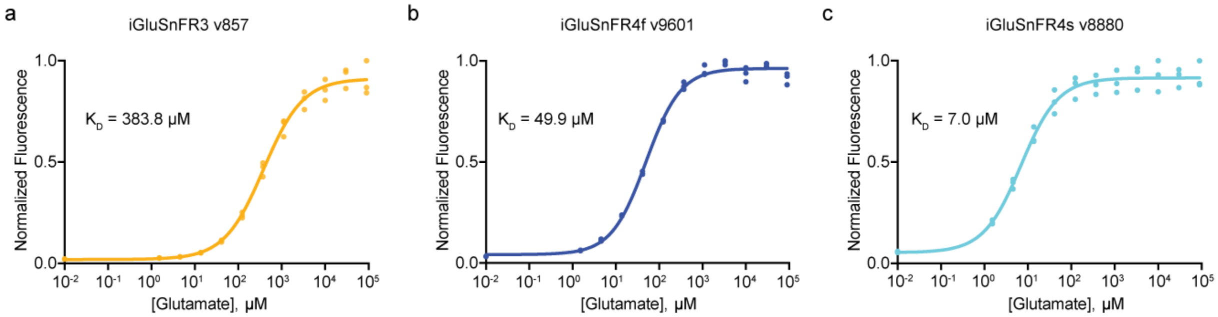

Supplementary Figure 2: Glutamate titration of purified soluble proteins.

a) iGluSnFR3.v857 (left), **b)** iGluSnFR4f (middle) and **c)** iGluSnFR4s (right) (pH 7.3 for all), with corresponding fits and dissociation constants (K_D). N = 3 titration series of a single protein sample for each variant.

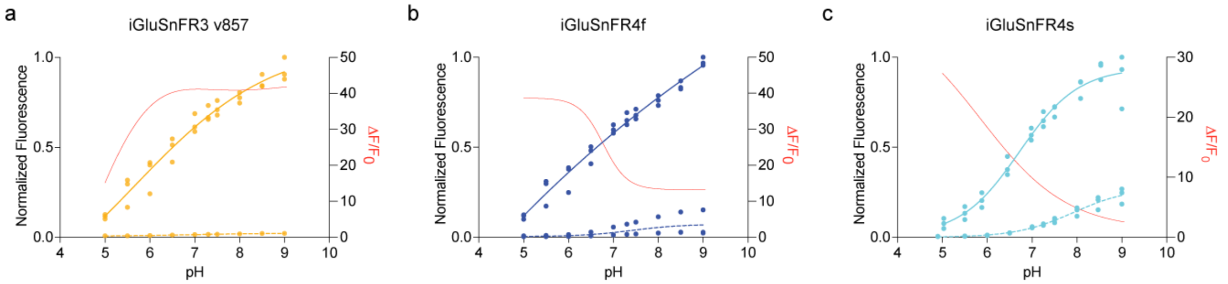

Supplementary Figure 3: pH titration of purified soluble proteins.

Fluorescence, normalized to minimum and maximum observed, at varying pH for **a)** iGluSnFR3.v857, **b)** iGluSnFR4f and **c)** iGluSnFR4s. Solid lines: saturating glutamate (10 mM, pH 7.3 buffered in PBS); Dotted lines: absence of glutamate. Sigmoidal fits are overlaid. Red lines show the fold change for +Glutamate vs -Glutamate conditions. All measurements were made using purified soluble protein; N = 3 titration series of a single protein sample for each measurement.

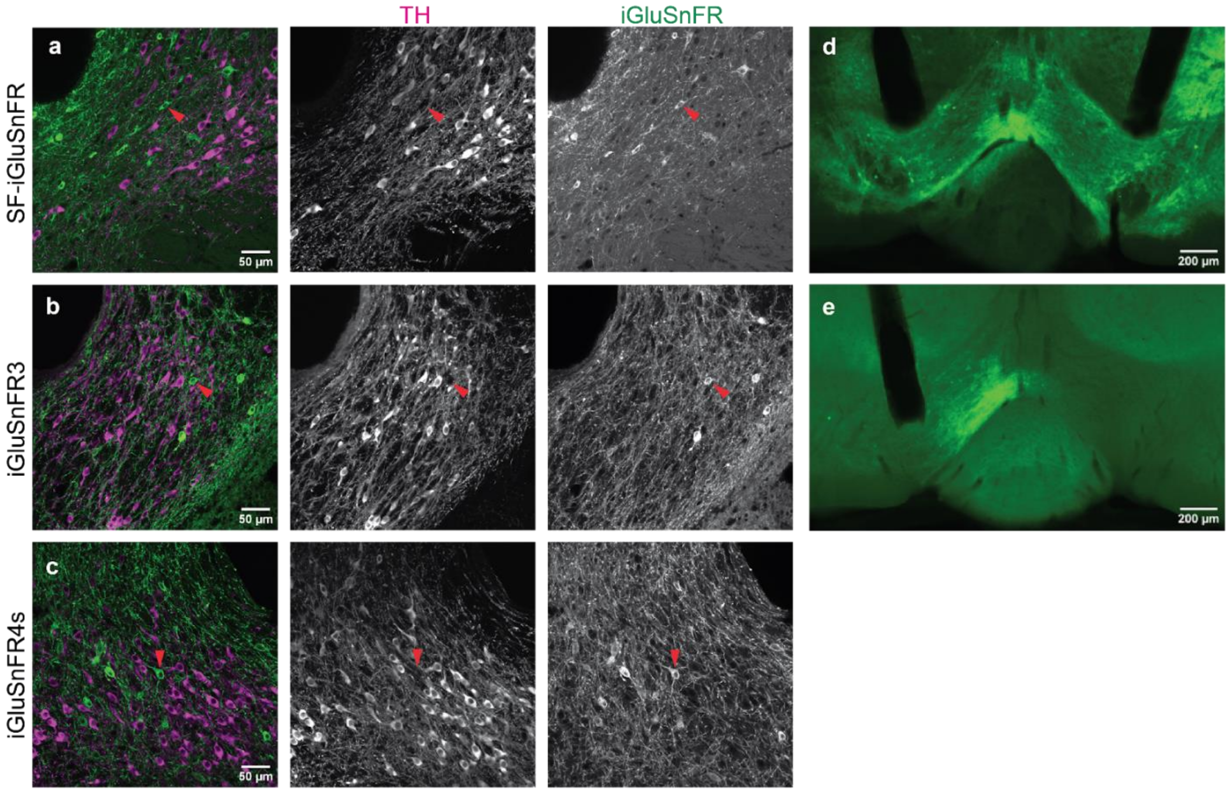

Supplementary Figure 4. Histological validation of fiber placement and iGluSNFR expression in fiber photometry experiments. Representative example confocal images used to verify fiber placement and expression, for SF-iGluSNFR (A), iGluSNFR3 (B), iGluSNFR4 (C). Red arrows highlight iGluSNFR-positive cells (green) non-overlapping with TH positive cells (magenta). (D-E) Example slide scanner images depicting (D) fiber placements over right and left VTA, and (E) single-hemisphere injection and fiber placement showing that AAV expression is largely contained to injected hemisphere.

Supplementary Table 1: Sequences of variants selected for *in vivo* testing.

Variant name	Mutations (GltI-N cp-mVenus GltI-C)
iGluSnFR3 v857	Template
iGluSnFR4s (v8880)	Y31Q Q98F K271F N499V
iGluSnFR4f (v9601)	Y31Q Q98F S182V K271F N499V
v7895	Y31Q Q98F N499V
v8179	Y31Q Q34A Q98F N499V
v8360	Y31Q Q34A Q98F K271G Q418S N499V
v8376	Y31E Q34A Q98F K271G N499V
v8881	Y31Q A185N K271G N499V

Supplementary Table 2: X-ray crystallography data collection and refinement statistics.

Values in parentheses are for the highest resolution shell, as implemented in XDS.

	**iGluSnFR3 (PDB: 9FBU)**
Data collection	
Space group	$P2_12_11$
Unit-cell parameters	
a, b, c (Å)	68.76, 100.84, 73.29
a, b, g (°)	90.00, 90.00, 90.00
Radiation source	ID23-1, ESRF
Wavelength (Å)	0.88560
Temperature (K)	100
Resolution range (Å)	50-1.70 (1.80-1.70)
No. of observed reflections	359919 (54172)
No. of unique reflections	56404 (8736)
Multiplicity	6.4 (6.2)
Completeness (%)	99.3 (98.8)
R_{merge} (%)	7.4 (64.6)
<I/s(I)>	14.6 (3.2)
$CC_{1/2}$ (%)[#]	99.8 (80.9)
Refinement	
Molecules per a.u.	1
No. of reflections	56401
No. of reflections in test set	2820
Resolution range (Å)	41.54-1.70

No. of non-hydrogen atoms	
Protein	3997
Ligand/ion	21
Water	334
Total	4352
R (%)	18.28
R_{free} (%)	21.07
RMS deviations from ideal	
bonds (Å)	0.007
angles (°)	0.921
B-factors (Å^2)	
Protein	26.38
Ligand/ion	21.33
Water	32.39
Average	26.81
Wilson B (Å^2)	23.12
Ramachandran statistics (%)	
favored regions	98.2
allowed regions	1.8
disallowed regions	0
Clashscore	1.88
