# Supplementary material for: Glutamate indicators with increased sensitivity and tailored deactivation rates
Source: Nat Methods. 2025 Dec 23;23(2):417–25. doi: 10.1038/s41592-025-02965-z (PMC12904790; doi:10.1038/s41592-025-02965-z)
Supplement: Supplementary file 2 — Reporting Summary [file 41592_2025_2965_MOESM2_ESM.pdf]

nature portfolio

Corresponding author(s): Kaspar Podgorski

Last updated by author(s): Oct 12, 2025

# Reporting Summary

Nature Portfolio wishes to improve the reproducibility of the work that we publish. This form provides structure for consistency and transparency in reporting. For further information on Nature Portfolio policies, see our Editorial Policies and the Editorial Policy Checklist.

## Statistics

For all statistical analyses, confirm that the following items are present in the figure legend, table legend, main text, or Methods section.

| n/a | Confirmed | |
|---|---|---|
| ☐ | ☒ | The exact sample size ($n$) for each experimental group/condition, given as a discrete number and unit of measurement |
| ☐ | ☒ | A statement on whether measurements were taken from distinct samples or whether the same sample was measured repeatedly |
| ☐ | ☒ | The statistical test(s) used AND whether they are one- or two-sided<br>*Only common tests should be described solely by name; describe more complex techniques in the Methods section.* |
| ☐ | ☒ | A description of all covariates tested |
| ☐ | ☒ | A description of any assumptions or corrections, such as tests of normality and adjustment for multiple comparisons |
| ☐ | ☒ | A full description of the statistical parameters including central tendency (e.g. means) or other basic estimates (e.g. regression coefficient) AND variation (e.g. standard deviation) or associated estimates of uncertainty (e.g. confidence intervals) |
| ☐ | ☒ | For null hypothesis testing, the test statistic (e.g. $F$, $t$, $r$) with confidence intervals, effect sizes, degrees of freedom and $P$ value noted<br>*Give P values as exact values whenever suitable.* |
| ☒ | ☐ | For Bayesian analysis, information on the choice of priors and Markov chain Monte Carlo settings |
| ☐ | ☒ | For hierarchical and complex designs, identification of the appropriate level for tests and full reporting of outcomes |
| ☐ | ☒ | Estimates of effect sizes (e.g. Cohen's $d$, Pearson's $r$), indicating how they were calculated |

*Our web collection on statistics for biologists contains articles on many of the points above.*

## Software and code

Policy information about availability of computer code

| | |
|---|---|
| Data collection | Custom MATLAB and Python scripts were used for high-throughput imaging analysis, electrophysiology, and fluorescence quantification. Software tools included MATLAB (versions 2021b through 2024b, as reported in the Methods), Python 3.9 (NumPy, SciPy, matplotlib), and ScanImage 2023 for microscope control. The GENIE project team used custom code for data acquisition (lightly modified from Wardill et al 2013; available from the GENIE project team upon request). Widefield imaging used WaveSurfer v1.0.6 and Hamamatsu HCImage 4.3.1, and NIS-Elements 4.1. |
| Data analysis | Data analysis was performed using custom Python and MATLAB code. GLM models were fitted using statsmodels (v0.13.5) in Python. Imaging data was processed with Suite2P and NMF-based algorithms. Code used for processing and analysing data is available in the code and data supplement (doi: 10.25378/janelia.30251743). |

For manuscripts utilizing custom algorithms or software that are central to the research but not yet described in published literature, software must be made available to editors and reviewers. We strongly encourage code deposition in a community repository (e.g. GitHub). See the Nature Portfolio guidelines for submitting code & software for further information.

# Data

Policy information about availability of data

All manuscripts must include a data availability statement. This statement should provide the following information, where applicable:

- Accession codes, unique identifiers, or web links for publicly available datasets
- A description of any restrictions on data availability
- For clinical datasets or third party data, please ensure that the statement adheres to our policy

All plasmids, constructs, and sequences used in this study are publicly available on Addgene: https://www.addgene.org/browse/article/28252906/

Structural data and the iGluSnFR3-based model used for iGluSnFR4 engineering are available via the RCSB Protein Data Bank under accession code 9FBU. https://www.rcsb.org/structure/9FBU

# Research involving human participants, their data, or biological material

Policy information about studies with human participants or human data. See also policy information about sex, gender (identity/presentation), and sexual orientation and race, ethnicity and racism.

| | |
|---|---|
| Reporting on sex and gender | *Use the terms sex (biological attribute) and gender (shaped by social and cultural circumstances) carefully in order to avoid confusing both terms. Indicate if findings apply to only one sex or gender; describe whether sex and gender were considered in study design; whether sex and/or gender was determined based on self-reporting or assigned and methods used.<br>Provide in the source data disaggregated sex and gender data, where this information has been collected, and if consent has been obtained for sharing of individual-level data; provide overall numbers in this Reporting Summary. Please state if this information has not been collected.<br>Report sex- and gender-based analyses where performed, justify reasons for lack of sex- and gender-based analysis.* |
| Reporting on race, ethnicity, or other socially relevant groupings | *Please specify the socially constructed or socially relevant categorization variable(s) used in your manuscript and explain why they were used. Please note that such variables should not be used as proxies for other socially constructed/relevant variables (for example, race or ethnicity should not be used as a proxy for socioeconomic status).<br>Provide clear definitions of the relevant terms used, how they were provided (by the participants/respondents, the researchers, or third parties), and the method(s) used to classify people into the different categories (e.g. self-report, census or administrative data, social media data, etc.)<br>Please provide details about how you controlled for confounding variables in your analyses.* |
| Population characteristics | *Describe the covariate-relevant population characteristics of the human research participants (e.g. age, genotypic information, past and current diagnosis and treatment categories). If you filled out the behavioural & social sciences study design questions and have nothing to add here, write "See above."* |
| Recruitment | *Describe how participants were recruited. Outline any potential self-selection bias or other biases that may be present and how these are likely to impact results.* |
| Ethics oversight | *Identify the organization(s) that approved the study protocol.* |

Note that full information on the approval of the study protocol must also be provided in the manuscript.

# Field-specific reporting

Please select the one below that is the best fit for your research. If you are not sure, read the appropriate sections before making your selection.

☒ Life sciences ☐ Behavioural & social sciences ☐ Ecological, evolutionary & environmental sciences

For a reference copy of the document with all sections, see nature.com/documents/nr-reporting-summary-flat.pdf

# Life sciences study design

All studies must disclose on these points even when the disclosure is negative.

| | |
|---|---|
| Sample size | No statistical method was used to pre-determine sample size. Sample sizes were matched to prior work with iGluSnFR3 and other fluorescent indicators, which were previously sufficient to detect differences in key metrics such as $\Delta F/F_0$, signal-to-noise ratio (SNR), and kinetic parameters. All main results were observed consistently across replicates. |
| Data exclusions | Data were excluded from analysis only on the basis of technical failures during data acquisition. |
| Replication | All key findings, including in vitro and in vivo indicator performance, were successfully replicated in at least two independent biological replicates. No major findings failed replication. |
| Randomization | Samples and animals were randomized across conditions to minimize bias. For in vitro screening, iGluSnFR4 variants were distributed across wells, and the layout was randomized across plates to control for positional and batch effects. For in vivo imaging, mice were randomly assigned indicator constructs at the time of AAV injection. Imaging regions (e.g., dendrites or boutons) were selected without knowledge of |

| | |
|---|---|
| | expected outcomes. Randomization was maintained throughout data acquisition and analysis pipelines to ensure fair comparisons between variants. |
| Blinding | The only experiment that involved blinding was the manual annotation of spine survival. In all other cases, blinding was unnecessary because data collection used highly automated instruments and/or clearly defined protocols that minimize the risk of experimenter bias. The benefit of blinding would not warrant the additional complexity and potential errors associated with it in this context. Similarly, the analyses performed were virtually all automated, making blinding irrelevant. Manual annotation was necessary to identify synaptic structures in two-photon volumes; annotations were not performed blinded, but were subsequently assessed by an expert blinded to indicator identity before analysis. Examples shown in figures were either randomly selected or manually selected as representative examples typical of the experiment. |

# Reporting for specific materials, systems and methods

We require information from authors about some types of materials, experimental systems and methods used in many studies. Here, indicate whether each material, system or method listed is relevant to your study. If you are not sure if a list item applies to your research, read the appropriate section before selecting a response.

## Materials & experimental systems

| n/a | Involved in the study |
|---|---|
| ☒ | ☐ Antibodies |
| ☒ | ☐ Eukaryotic cell lines |
| ☒ | ☐ Palaeontology and archaeology |
| ☐ | ☒ Animals and other organisms |
| ☒ | ☐ Clinical data |
| ☒ | ☐ Dual use research of concern |
| ☒ | ☐ Plants |

## Methods

| n/a | Involved in the study |
|---|---|
| ☒ | ☐ ChIP-seq |
| ☒ | ☐ Flow cytometry |
| ☒ | ☐ MRI-based neuroimaging |

## Animals and other research organisms

Policy information about studies involving animals; ARRIVE guidelines recommended for reporting animal research, and Sex and Gender in Research

| | |
|---|---|
| Laboratory animals | Mice: Emx1-cre (JAX 005628), female, 8–30 wks; C57BL/6, mixed sexes, 8–30 wks; Scnn1a-Tg3-Cre (JAX 009613), male, 8–30 wks |
| Wild animals | The study did not involve wild animals. |
| Reporting on sex | Both male and female mice were used in this study. No sex-specific differences were observed in results, and data from both sexes were pooled. |
| Field-collected samples | The study did not involve field-collected samples. |
| Ethics oversight | All animal procedures were conducted in accordance with institutional animal care and use protocols approved by the Allen Institute for Neural Dynamics, Janelia Research Campus of HHMI, the University of California San Diego, Technical University of Munich, University College London, and St. Jude Children's Research Hospital. All procedures followed the relevant national and institutional guidelines for the care and use of laboratory animals.<br><br>Allen Institute for Neural Dynamics: Protocol 2109<br>HHMI Janelia Research Campus: Protocols 19-176, 22-0214.01<br>University of California, San Diego: Protocol S02174M<br>Technical University of Munich: Protocols 2532.Vet_02-23-24 and 2532.Vet_02-21-121<br>University College London: Animal License PP3929312<br>St. Jude Children's Research Hospital: Protocol no. 3193 |

Note that full information on the approval of the study protocol must also be provided in the manuscript.

## Plants

Seed stocks

*Report on the source of all seed stocks or other plant material used. If applicable, state the seed stock centre and catalogue number. If plant specimens were collected from the field, describe the collection location, date and sampling procedures.*

Novel plant genotypes

*Describe the methods by which all novel plant genotypes were produced. This includes those generated by transgenic approaches, gene editing, chemical/radiation-based mutagenesis and hybridization. For transgenic lines, describe the transformation method, the number of independent lines analyzed and the generation upon which experiments were performed. For gene-edited lines, describe the editor used, the endogenous sequence targeted for editing, the targeting guide RNA sequence (if applicable) and how the editor was applied.*

Authentication

*Describe any authentication procedures for each seed stock used or novel genotype generated. Describe any experiments used to assess the effect of a mutation and, where applicable, how potential secondary effects (e.g. second site T-DNA insertions, mosiacism, off-target gene editing) were examined.*
